# Information entropy dynamics, self-organization, and cybernetical neuroscience

**DOI:** 10.3389/fnetp.2025.1539166

**Published:** 2025-03-21

**Authors:** Alexander Fradkov

**Affiliations:** Control of Complex Systems Lab, Institute for Problems of Mechanical Engineering, Saint Petersburg, Russia

**Keywords:** information, entropy, network, speed-gradient, evolution, self-organization, control, network physiology

## Abstract

A version of the speed-gradient evolution models for systems obeying the maximum information entropy principle developed by H. Haken in his book of 1988 is proposed in this article. An explicit relation specifying system dynamics for general linear constraints is established. Two versions of the human brain entropy detailed balance-breaking model are proposed. In addition, the contours of a new scientific field called *cybernetical neuroscience* dedicated to the control of neural systems have been outlined.

## 1 Introduction

In Haken (1988), the eminent scientist Hermann Haken explored the interplay between the concepts of information and self-organization. He took a significant step toward broadening the applicability of the Gibbs–Jaynes principle of maximum entropy (Jaynes, 1957). Specifically, Haken incorporated functions that act as order parameters in nonequilibrium phase transitions into the set of additional constraints. In Chapter 3 of the book, he presents a modified version of the maximum entropy principle. Haken’s adaptation of this principle involves seeking a new future state of the system that maximizes information while adhering to physical conditions describing the system’s physical properties.

Let the elements of some system, for example, molecules of an ideal gas, stay in n cells. If we are looking for the distribution of molecules by possible states (cells), that is, we need to find the probabilities $p_{1},p_{2},\ldots ,p_{n}$ where $p_{i}$ is the probability of finding a molecule in a cell i . Then, the information entropy S of the system is defined as

$$S=-K\overset{n}{\underset{i=1}{\sum }}p_{i}\ln p_{i},$$ (1)

where K is the Boltzmann constant.

The physical conditions act as constraints; for example, the position of the center of masses may be given:

$$\overset{n}{\underset{i=1}{\sum }}p_{i}q_{i}=M,$$ (2)

where $q_{i}$ is the position of the i th cell, the value M is the coordinate of the center of masses, and N is the total number of the particles. Alternatively, let $f_{i}$ be the kinetic energy of the i th particle. Then, the average value of the kinetic energy of the system may be specified by

$$\overset{n}{\underset{i=1}{\sum }}p_{i}f_{i}=E,$$

and so on. In many important cases, all the constraints specify the values $L_{k}$ of some linear combinations of some characteristics $f_{i}^{k},\;k=1,\ldots ,L$ of the system:

$$\overset{n}{\underset{i=1}{\sum }}p_{i}f_{i}^{k}=L_{k},$$

Additionally, the normalization constraint for the distribution should be added:

$$\overset{n}{\underset{i=1}{\sum }}p_{i}=1,p_{i}\geq 0.$$ (3)

Several examples in Haken (1988) illustrate the form of the system’s state distribution that achieves maximum information, as well as the state corresponding to the self-organization of the system. However, neither in Haken’s book nor in any other known works does the question arise regarding how a system evolves to attain the state of maximum information entropy (or self-organized state).

The question “How does a system evolve when striving for a state of maximum entropy?” was first addressed in the works by Fradkov (2007) and Fradkov (2008) and further investigated in a series of subsequent publications. It was hypothesized that this evolution aligns with the speed-gradient principle, originally developed within control systems theory, as seen in Fradkov (1979), Fradkov (1991), and Fradkov et al. (1999). According to this hypothesis, the evolution process can be understood if the system aims to maximize a certain functional. If the system seeks to achieve the optimal state, it should logically strive to do so in the most efficient manner.

Indeed, if the objective is to increase the value of a given target functional, the quickest path to achieving this goal would be to follow the direction of the speed gradient: the gradient of the rate at which this functional changes. Subsequent studies have examined the application of the speed-gradient principle to different types of entropies, including Shannon, Rényi, Tsallis, relative entropy, and Kullback–Leibler divergence (Fradkov, 2008; Fradkov and Shalymov, 2014; Fradkov and Shalymov, 2015; Shalymov et al., 2017). The evolution of distributed systems governed by the law of maximum differential entropy was also analyzed by Fradkov and Shalymov (2015a). In each instance, it was demonstrated and mathematically verified that the trajectories of systems evolving according to the speed-gradient principle converge to a state of maximum entropy that is asymptotically stable.

Herein, we show that analogous principles and laws govern the transition to the state of maximum information entropy described in Hermann Haken’s works. We propose an explicit form for the speed-gradient evolution of systems characterizing the dynamics of information entropy in the presence of multiple linear constraints, as discussed in Section 3.2 of Haken (1988). This case generalizes previous considerations involving constraints related to the conservation of mass and energy.

## 2 Principle of maximum entropy and speed-gradient formalism

Consider the problem of finding the system dynamics equations in the form

$$dx/dt=F\left(x,u,t\right),t\geq 0,$$ (4)

where x is the system state vector, and u is input vector. The above-mentioned *speed-gradient principle* is formulated as follows (Fradkov, 1991; Fradkov, 2007). Among all possible motion of (Equation 4), only those are realized for which the input vector u(t) changes proportionally to the gradient in u of the speed of changing in t of an appropriate goal functional $Q_{t}$ . If constraints are imposed on the system motion, then the speed-gradient vector should be projected onto the set of admissible (compatible with constraints) directions.

Consider the formalism developed in Chapter 3 of Haken’s book as an interpretation of Jaynes’ maximum entropy principle for the discrete systems possessing information entropy

$$S\left(p\right)=-K\overset{n}{\underset{i=1}{\sum }}p_{i}\;ln\;p_{i},$$ (5)

where $p_{i},\;i=1,2,\ldots ,n$ are the probabilities or relative frequencies for staying of the particle in the i th state, n is the total number of particles, K is the Boltzmann constant, and S(p) is the information or information entropy of the state.

Haken writes that the main task to which the book is devoted is to find ways to determine the frequencies $p_{i}$ , taking into account the constraints and available additional information. For example, when considering an ideal one-dimensional gas, one can measure, for example, the position of the center of mass having the form (Equation 2), where $q_{i}$ is the i -th cell position. In addition, the normalization condition (Equation 3) should be valid.

According to the principle of maximum entropy, the distribution that carries the greatest information is realized with the greatest probability. This also happens in other cases when the maximum entropy principle is applicable.

Let us pose the question: how and in what way does the system move to a state with maximum information? In order to find an affirmative answer, it is necessary to formulate the problem more formally, as finding the system dynamics in the form

dp/dt=u, (6)

where $p=col\left(p_{1},p_{2},\ldots ,p_{n}\right)$ is the column^1^ vector of the system state distribution, and u is the external input vector-function to be determined.

Assume that the following constraints hold during system evolution:

Ap=b, (7)

where A is the m×n− matrix, b is the m -vector, and m is the number of constraints. Assume that matrix A has full rank; that is, different constraints are linearly independent. Let the constraints (Equation 7) be valid in the initial time instant: Ap(0)=b . Choose entropy S(p) defined in Equation 5 as the goal function. According to the speed-gradient principle, among the whole set of the directions (ways) of evolution satisfying constraints (Equation 7), the one that is realized is the movement along the trajectory of the fastest growth of the entropy S(p(t)) . In other words, according to the speed-gradient principle, the system takes the path of maximum energy production.

Such a statement allows one to determine the input vector-function u(p) explicitly. To this end, evaluate the projection onto the set of the points satisfying constraints (Equation 7) of the gradient in u of the speed of change of goal function - entropy (Equation 5) along trajectories of Equation 6. Apparently, the speed of change of the goal function along Equation 6 is as follows: $\dot{S}\left(p,u\right)=\nabla_{p}S{\left(p\right)}^{T}u$ , and the gradient of this speed in u is ∇S(p) , where ∇ denotes the gradient (vector of partial derivatives of a function, and upper index T means transposition. However, one needs to take into account the constraints (Equation 7), that is, to make a projection onto the set P∗={p: Ap=b} . Introducing and evaluating the m -vector of Lagrange multipliers $\lambda_{1},\ldots ,\lambda_{m}$ and taking into account initial conditions Ap(0)=b , the following expression is obtained after some algebra:

$$u=\gamma \left[I_{n}-A^{T}{\left(AA^{T}\right)}^{-1}A\right]\nabla_{p}S\left(p\right),$$ (8)

where γ>0 is the gain parameter, and $I_{n}$ is the unity n×n matrix. It is seen that the matrix in the square brackets in (Equation 8) is nothing but the matrix $P_{A}$ of the projection onto the subspace determined by the condition Ap=0 .

Recall that in our case, the goal function is entropy S in Equation 1. Its partial derivatives are defined as follows: $\partial S/\partial p_{i}=ln\left(p_{i}\right)+1$ . Therefore, the system will evolve according to the rule

$$dp/dt=\gamma P_{A}\left(ln\left(p\right)+1\right),$$ (9)

where 1 is the m -vector with all components equal to 1. Note that the conditions $p_{i}>0$ , which are necessary for keeping the system well posed, will be valid automatically for solutions of (Equation 9), because they are valid in the initial time instant, and the goal function S(p) grows to ∞ when any $p_{i}$ tends to 0.

The above results can be extended to take into account the topology of the network describing interactions of the nodes (Fradkov et al., 2016).

## 3 Application to network physiology problems

Recently, a new multi-disciplinary research field on the border between system science and biology entitled *network physiology* has emerged. It is devoted to the study of biological and physiological systems possessing network structures (Ivanov and Bartsch, 2014; Ivanov, 2021). It is well known that both animal and human organisms are integrated networks, where multi-component physiological systems, each with its own regulatory mechanism, continuously interact to coordinate their functions. However, we still do not know the principles and mechanisms through which diverse systems and sub-systems in the human body dynamically interact as a network and integrate their functions to generate physiological states in health and disease. Network physiology aims to address these fundamental questions.

Among the tasks of network physiology are those that are similar to the tasks of information dynamics and self-organization considered in the works of Hermann Haken, for example, Haken (1988). For example, as is known, the entropy of a working brain can be measured using fMRI equipment (Lynn et al., 2021). Therefore, it is technically possible to use the concept of entropy in the analysis of the work of the human brain. Indeed, the neurons and the neuron ensembles of the human brain can stay in different states and change their states in time. Such an uncertainty can be described by some probabilities, and then the entropy of the state of the brain at each time instant can be evaluated. Therefore, the entropy production can also be evaluated. Hence, the results of the previous section that propose the principle to estimate dynamics of the information entropy changes can be used to analyze the state and dynamics of the real brain.

Indeed, the analysis of the whole-brain imaging data has demonstrated that the human brain breaks detailed balance at large scales and that the brain’s entropy production (that is, its distance from detailed balance) varies critically with the specific function being performed, increasing with both physical and cognitive demands (Lynn et al., 2021). To analyze the mutual dynamics of the regions of the spatially distributed system, a network-adopted version of the speed-gradient principle (Fradkov et al., 2016) can be employed.

Among other examples related to network physiology problems, one can mention analysis of the interactions among brain and cardiac networks (González et al., 2022), spike-timing-dependent plasticity and its role in Parkinson’s disease pathophysiology (Madadi Asl et al., 2022), and criticality in the healthy brain (Shi et al., 2022).

## 4 Dynamics of human brain entropy

As an example, let us consider the process of breaking and restoring detailed balance between regions in the human brain (Lynn et al., 2021). This process is interesting because, as was noted in the celebrated work by Schrödinger (1944), see also Gnesotto et al. (2018), the brain, as well as a living being as a whole, tends to increase its entropy. At first glance, the number of neurons in the brain is overwhelmingly large, and the structure of connections between them is overwhelmingly complex, making comprehensive analysis impractical. However, recent achievements of the international “Connectome” project have demonstrated that for many purposes, describing the brain as a network with a finite and relatively small number of nodes (approximately 100) suffices; see Van Essen et al. (2013). Hence, coarse-grained models of the human brain can be constructed that maintain manageable complexity. While at rest, the brain sustains a detailed balance of transitions between states. When engaged in physical or cognitive tasks, however, this detailed balance breaks down. Given that information entropy serves as a measure of uncertainty, modeling brain dynamics seems natural on its basis.

Based on the hypothesis that the brain, and perhaps the entire living organism, strives to break the detailed balance and increase information entropy, it is interesting to find the law or model according to which the brain increases its entropy. It seems plausible that the brain (or organism) endeavors to maximize its entropy in an optimal way. How might this be achieved? Drawing upon prior discussions in Section 2, we propose addressing this issue through the speed-gradient principle.

Consider a system with the state vector $x_{t}$ at time t , which can take N possible values, denoted as {1,2,…,N} . Suppose that the dynamics of the system are stochastic and let $P_{ij}\left(t\right)$ be the probability of an event $\left\{x_{t-1}=i,x_{t}=j\right\}$ . In other words, $P_{ij}\left(t\right)$ are forward transition probabilities, and $P_{ji}\left(t\right)$ are backward transition probabilities. If the system has Markovian dynamics [e.g., Ising model, see Lynn et al. (2021)], then the rate of changing entropy (entropy production) is given by Lynn et al. (2021):

$$\dot{S}\left(t\right)=\overset{N}{\underset{i,j=1}{\sum }}P_{ij}\left(t\right)\log\frac{P_{ij}\left(t\right)}{P_{ji}\left(t\right)},$$ (10)

Evidently, the right-hand side of Equation 10 corresponds to Kullback–Leibler divergence measuring the distance between two distributions (forward and backward movements). If the system is in the state of the detailed balance, then $P_{ij}=P_{ji}$ ; that is, the entropy production vanishes and *vice versa*, that is, $\dot{S}\left(t\right)$ is a measure of broken detailed balance. Therefore, the problem is to find the law of changing $P_{ij},P_{ji}$ , such that the entropy growth as fast as possible under normalization constraints

$$\overset{N}{\underset{j=1}{\sum }}P_{ij}=1,\;i=1,\ldots ,N.$$ (11)

$$P_{ij}\geq 0,\;i,\;j=1,\ldots ,N.$$ (12)

Assume for simplicity that the reverse probabilities $P_{ji}$ are not changed: $P_{ji}=const$ and evaluate the gradients of the entropy production according to the approach of Section 2.

The gradients of the entropy production should be taken over the controlling (input) variables. As such, it is natural to take those probabilities that determine the next coarse-grained state of the brain numbered j , and the direction of the fastest growth of $\dot{S}\left(t\right)$ , that is, the gradient of $\dot{S}\left(t\right)$ with respect to those probabilities. We have a fixed current state, and i is the index corresponding to the current state. This means that we need to take the gradient with respect to the next state vector, which plays the role of a control action because the brain goes into a new state, and we want to know how this choice is made. Therefore, let us evaluate the gradient of $\dot{S}$ with respect to $P_{ij}$ , assuming that $P_{ji}$ are fixed. To avoid notational confusion, replace summation indices (i,j) with (k,l) . Then, the expression for $\dot{S}$ reads

$$\begin{array}{c} \frac{\partial }{\partial P_{ij}}\left(\dot{S}\left(t\right)\right)=\frac{\partial }{\partial P_{ij}}\overset{N}{\underset{k,l=1}{\sum }}P_{kl}\log\frac{P_{kl}}{P_{lk}}. \end{array}$$ (13)

Note that k,l in (Equation 13) are running indices, and for any fixed pair (i,j) , only one term in the sum (Equation 13) depends on $P_{ij}$ . Hence

$$\begin{array}{c} \frac{\partial }{\partial P_{ij}}\left(\dot{S}\left(t\right)\right)=1\cdot \log\frac{P_{ij}}{P_{ji}}+P_{ij}\frac{\partial }{\partial P_{ij}}\left(log\left(P_{ij}\right)-log\left(P_{ji}\right)\right)=\\ \log\frac{P_{ij}}{P_{ji}}+1. \end{array}$$ (14)

To take into account constraints (Equation 11), introduce Lagrange multipliers $\lambda_{i},\;i=1,\ldots ,N$ and choose them in such a way that the equations

$${\dot{P}}_{ij}=\log\frac{P_{ij}}{P_{ji}}+1-\lambda_{i}.$$ (15)

satisfy constraints $\sum_{l=1}^{N}{\dot{P}}_{il}=0,\;i=1,\ldots ,N$ . Then, the constraints (Equation 11) will be valid for all t≥0 , provided that they are valid for t=0 . As for constraints (Equation 12), they will be fulfilled automatically for all t≥0 if they are strictly fulfilled for t=0 $\left(P_{ij\left(0\right)}>0\right)$ because $P_{ij}$ appears in Equation 15 under the log operation and cannot approach zero. It is easy to see that such $\lambda_{i}$ may be chosen as follows:

$$\lambda_{i}=1+\frac{1}{N}\overset{N}{\underset{l=1}{\sum }}\log\frac{P_{il}}{P_{li}}$$

Finally, the law of the fastest transition probabilities evolution is as follows

$${\dot{P}}_{ij}\left(t\right)=\gamma \left(\log\frac{P_{ij}\left(t\right)}{P_{ji}}-\frac{1}{N}\overset{N}{\underset{l=1}{\sum }}\log\frac{P_{il}\left(t\right)}{P_{li}}\right),i,\;j=1,\ldots N,$$ (16)

where γ>0 is the gain (activity) coefficient. Because in reality, only measurements in the discrete (sampled) time instants are possible, we arrive at the following final relation:

$$P_{ij}\left(t\right)-P_{ij}\left(t-1\right)=\gamma \left(\log\frac{P_{ij}\left(t-1\right)}{P_{ji}}-\frac{1}{N}\overset{N}{\underset{l=1}{\sum }}\log\frac{P_{il}\left(t-1\right)}{P_{li}}\right),i,\;j=1,\ldots ,N.$$ (17)

Let us consider a modified version of the breaking detailed balance model based on the same speed-gradient principle. Once again, we start with the assumption that entropy production grows in the optimal manner. However, let us now measure the entropy production by its deviation from the uniform distribution corresponding to the maximum system entropy. It means that the uniform distribution is chosen as the base level for the entropy production evaluation, and the following model is used instead of Equation 10:

$$\dot{S}\left(t\right)=\overset{N}{\underset{k,l=1}{\sum }}P_{kl}\left(t\right)\log\frac{P_{kl}\left(t\right)}{P^{*}},$$ (18)

where probability $P^{*}=\frac{1}{N^{2}}$ defines the uniform distribution.

The Kullback–Leibler divergence (Equation 18) can also serve as a measure of the current state deviation from the detailed balance state. Taking Equation 18 as the model of the goal function for the speed-gradient method and repeating the calculations, we obtain the following expressions instead of Equation 16:

$${\dot{P}}_{ij}\left(t\right)=\gamma \left(\log\frac{P_{ij}\left(t\right)}{P^{*}}-\frac{1}{N}\overset{N}{\underset{l=1}{\sum }}\log\frac{P_{il}\left(t\right)}{P^{*}}\right),i,\;j=1,\ldots ,N.$$ (19)

where γ>0 is the gain (activity) coefficient. Because in reality, only measurements in the discrete (sampled) time instants are possible, we arrive at the following final relation instead of Equation 17:

$$P_{ij}\left(t\right)-P_{ij}\left(t-1\right)=\gamma \left(\log\frac{P_{ij}\left(t-1\right)}{P^{*}}-\frac{1}{N}\overset{N}{\underset{l=1}{\sum }}\log\frac{P_{il}\left(t-1\right)}{P^{*}}\right),i,\;j=1,\ldots ,N.$$ (20)

Equations 16, 19 and Equations 17, 20 are continuous-time models and discrete-time models, respectively, for human brain entropy dynamics proposed via speed-gradient method. Which model is closer to reality? It is necessary to conduct a series of experiments with real data to answer this question.

## 5 Networks and cybernetical neuroscience

Over the past 2 decades, system theory and cybernetics have yielded many new results and approaches that enable researchers to study various properties of complex networks. These findings can be applied to network physiology. For instance, numerous stability and synchronization criteria for complex networks are relevant to network models composed of interconnected mathematical models of neurons. The first results on the control of neuron and neural network models were obtained in the 1990s, focusing on chaos control and synchronization. Carroll (1995) proposed an algorithm for pulse synchronization control of two FitzHugh–Nagumo (FHN) neuron models, drawing parallels between neuronal and electrical processes. In Dragoi and Grosu (1998), an algorithm was designed to control a chain of FHN neurons, aiming to synchronize each neuron oscillation with those of a “reference” neuron. The stability of the synchronization process was established within a certain range of initial conditions using a linear approximation.

Plotnikov et al. (2016a) proposed algorithms for synchronizing a heterogeneous network of diffusion-coupled models of FHN neurons with a hierarchical architecture based on the speed-gradient method. Synchronization conditions were obtained based on the Lyapunov function method. Similar results were obtained for adaptive control algorithms that do not require precise knowledge of the neuron model parameters (Plotnikov et al., 2016b).

The Lyapunov function method and the speed-gradient method have also been effectively utilized in designing and analyzing control algorithms for synchronization and chaos control problems involving Hindmarsh–Rose models and their networks (Plotnikov, 2021; Semenov et al., 2022).

Currently, a growing body of research focuses not only on studying the properties of neural networks with neurophysiological interpretations but also delves deeply into the challenges associated with intentionally creating or eliminating these properties, that is, controlling networks. Other cybernetics-related tasks concerning networks of neurons or their models are being explored as well, such as state and parameter estimation, pattern recognition, and machine learning. In summary, there is a notable trend leading to the establishment of a substantial and significant new domain within computational neuroscience, which can naturally be called *cybernetical neuroscience*.

The main directions of the research in cybernetical neuroscience are as follows (Fradkov, 2024):

1. Analysis of the conditions for the models of neural ensembles to possess some special behaviors observed in the brain, such as synchronization, desynchronization, spiking, bursting, solitons, chaos, and chimeras.
2. Synthesis of external (control) inputs that create the special behaviors in the brain models.
3. Estimation of the state and parameters of the brain models based on the results of measuring input and output variables.
4. Classification of brain states and human intentions (using adaptation and machine learning methods) based on real brain state measurements (invasive or noninvasive).
5. Design of control algorithms that provide specified properties of a closed loop system consisting of a controlled neural system and a controlling device, interacting via brain-computer interface.

The approach to searching for how a system should evolve to reach the state of maximum information entropy presented in this article also originated in the area of control science or cybernetics. Its applications to neural systems belong to the area of cybernetical neuroscience.

## 6 Conclusion

This article proposes a version of the speed-gradient evolution model for systems following the maximum information entropy principle developed by H. Haken in his seminal book in 1988. An explicit relationship (Equation 8) defining system dynamics for general linear constraints Ap−b=0 is derived. Analogous results can be formulated for the spatially continuous case, where discrete information entropy is replaced by differential information entropy, in line with Fradkov and Shalymov (2015a). The approach is also extended to living systems. Two versions of a human brain entropy detailed balance-breaking model are proposed (Equations 16, 17, 19, 20).

Furthermore, the contours of a novel scientific area termed *cybernetical neuroscience*, focused on controlling neural systems, are delineated.

Future research might focus on examining the diverse dynamic issues in network physiology. For example, the methodology presented here could be applied to recent findings on utilizing entropy to analyze brain dynamics, as reported by Antonopoulos et al. (2015), Jirsa and Sheheitli, (2022), Keshmiri (2020), and Yufik (2019).

## Data Availability

The original contributions presented in the study are included in the article/Supplementary Material; further inquiries can be directed to the corresponding author.

## Footnotes

1 Notation col stands for column vector hereafter.
